# *N*-Acetylcysteine Reduced Ischemia and Reperfusion Damage Associated with Steatohepatitis in Mice

**DOI:** 10.3390/ijms21114106

**Published:** 2020-06-09

**Authors:** Natalie Chaves Cayuela, Marcia Kiyomi Koike, Jacqueline de Fátima Jacysyn, Roberto Rasslan, Anderson Romério Azevedo Cerqueira, Soraia Katia Pereira Costa, José Antônio Picanço Diniz-Júnior, Edivaldo Massazo Utiyama, Edna Frasson de Souza Montero

**Affiliations:** 1 Laboratory of Surgical Physiopathology—LIM-08, Department of Surgery, Faculdade de Medicina da Universidade de São Paulo (FMUSP), 01246-903 São Paulo, SP, Brazil; natalie.chaves@gmail.com (N.C.C.); jacysyn@gmail.com (J.d.F.J.); robertorasslan@uol.com.br (R.R.); edivaldo.utiyama@hc.fm.usp.br (E.M.U.); 2 Department of Emergency Medicine—LIM-51, FMUSP, 01246-903 São Paulo, SP, Brazil; mkoike2011@gmail.com; 3 General and Trauma Surgery Division, Department of Surgery, ICHC-FMUSP, 01246-903 São Paulo, SP, Brazil; 4 Departmento de Farmacologia, Instituto de Ciêncas Biomédicas, Universidade de São Paulo (USP), 05508-900 São Paulo, SP, Brazil; anderson.romerio@gmail.com (A.R.A.C.); skcosta@usp.br (S.K.P.C.); 5 Electron Microscopy Laboratory, Section of Hepatology, Instituto Evandro Chagas, 66093-020 Belém, PA, Brazil; joseantonio@iec.gov.br

**Keywords:** liver, mouse, steatohepatitis, cytokines, oxidative stress, antioxidant enzymes, cell death, acetylcysteine

## Abstract

*N*-acetylcysteine (NAC) is a pharmacological alternative with great potential for reducing the deleterious effects of surgical procedures on patients with steatohepatitis. We evaluated the effect of NAC on hepatic ischemia/reperfusion (I/R) injury in C57BL/6J mice, 8 weeks-old, weighing 25–30 g, with steatohepatitis induced by a methionine- and choline-deficient (MCD) diet. Groups: MCD group (steatohepatitis), MCD-I/R group (steatohepatitis plus 30 min of 70% liver ischemia and 24 h of reperfusion), MCD-I/R+NAC group (same as MCD-I/R group plus 150 mg/kg NAC 15 min before ischemia), and control group (normal AIN-93M diet). Liver enzymes and histopathology; nitrite and TBARS (thiobarbituric acid reactive substances) levels; pro-inflammatory cytokines; antioxidants enzymes; Nrf2 (nuclear factor erythroid-2-related factor 2) expression; and apoptosis were evaluated. In the group treated with NAC, reductions in inflammatory infiltration; AST (aspartate aminotransferase), nitrite, and TBARS levels; GPx (gutathione peroxidase) activity; cytokines synthesis; and number of apoptotic cells were observed while the GR (glutathione reductase) activity was increased. No differences were observed in Nfr2 expression or in SOD (superoxide dismutase), CAT (catalase), and GST (glutathione S-transferase) activities. Thus, it may be concluded that NAC exerts beneficial effects on mice livers with steatohepatitis submitted to I/R by reducing oxidative stress, inflammatory response, and cell death.

## 1. Introduction

Nonalcoholic fatty liver disease (NAFLD) is one of the most prevalent hepatic diseases in the global population [1,2], affecting 4 to 46% of individuals [3,4], and 90% of cases occur in morbidly obese individuals [1]. NAFLD is characterized by the presence of macroscopic steatosis in more than 5–10% of hepatocytes in the absence of other etiologies of hepatic diseases [1,5], such as excessive alcohol consumption, autoimmune or drug-induced diseases, or viral hepatitis [6]. The histological spectrum of NAFLD may range from simple steatosis to nonalcoholic steatohepatitis (NASH), with progression to liver fibrosis and cirrhosis [5,6,7].

NAFLD can increase liver susceptibility to hepatic ischemia/reperfusion (I/R) injury and has become the most important clinical risk factor for this type of liver injury [8,9,10], increasing the risk of postoperative morbidity and mortality, including cases of transplantation [11,12]. NAFLD is also strongly associated with primary graft dysfunction and low survival among transplant recipients as well as poor outcomes in procedures that require transient ischemia [13,14,15].

The mechanisms by which NAFLD and I/R cause damage in the liver are complex and involve several cellular components, factors, and mediators present during oxidative stress and inflammation, such as reactive oxygen species (ROS), reactive nitrogen species (RNS), antioxidant enzymes, transcription factors, cytokines, and neutrophil infiltration, amongst others. The oxidative stress is a result of the imbalance between free radicals production and the antioxidant mechanism. In NAFLD/NASH and I/R, free radicals, ROS, RNS, and others, produced mainly by Kupffer cells, act on hepatic cells causing lipid peroxidation in the cellular membrane, mitochondrial dysfunction, DNA damage, and may activate the cell death signaling pathway by necrosis and apoptosis. Then, cells of the immune system are recruited to the place of injury; resident innate immune cells and liver cells, through a nuclear factor κ-light-chain enhancer-activated B cells (NF-κB)-mediated mechanism, release pro-inflammatory cytokines that intensify the hepatic damage by increasing the production of ROS/RNS and activate the pro-apoptotic signal via a caspase cascade. Moreover, Kupffer cells amplify the inflammatory response through attraction and adhesion of neutrophils and mast cells, the clumping together of platelets, and obstruction of local microcirculation, all leading to decrease sinusoidal blood flow (ischemia) [16]. Cytokines derived from Kupffer cells, such as TGF-β, TNF-α, and IL-1, also may induce the hepatic stellate cells (HSC), previously activated by ROS, RSN, and malondialdehyde (MDA), to proliferate and transform into myofibroblasts, promoting increased production of collagen and hence causing fibrosis in the liver [17].

Endogenous antioxidants, such as glutathione (GSH), superoxide dismutase, catalase, and reduced glutathione-related enzymes, play a vital role in the maintenance of the oxidative/antioxidant balance by their capability to scavenge free radicals, contributing to a reduction in tissue damage [18]. Nrf2 is an important transcription factor that regulates this antioxidant defense system by maintaining cellular redox homeostasis, which allows adaptation and cell survival, and by mediating the gene expression that encodes detoxification and antioxidant enzymes. The inactivation of Nrf2 occurs through the bonding of regulator Kelch-like ECH-associated protein 1 (Keap1) that directs Nrf2 to its degradation by ubiquitination. However, the increase of ROS concentration during oxidative stress promotes the change of the Keap1 conformational state through the modification of the sulfhydride groups of the cysteine residues, and causes the dissociation of Nrf2 from this regulator. After, Nrf2 is translocated to the nucleus, where it bonds to a specific DNA sequence known as the antioxidant response element (ARE) and promotes the activation of genes associated with antioxidant and detoxifying responses, such as, glutathione S-transferase (GST) and NAD(P)H quinone oxidoreductase-1 (NQO1) [19,20].

One of the most important avenues of research in regard to treatment of hepatic diseases is into the attenuation of oxidative and inflammatory liver damage. To decrease oxidative stress and inflammation that leads to cell death in the liver, pharmacological therapy involving administration of exogenous antioxidants has recently been evaluated in animal models [21]. Among these antioxidants, *N*-acetylcysteine has great potential for reducing the deleterious effects of oxidative stress and inflammatory response in different types of diseases by acting on detoxification of ROS, inhibition the synthesis of NO, upregulation of antioxidant enzymes, immunomodulatory activity, and regulation of apoptosis [22]. In our laboratory, the effects of *N*-acetylcysteine (NAC) have been studied in various experimental models of I/R [23,24,25,26,27,28]. In a hemorrhagic shock model, NAC protected pulmonary tissue [23,24]. In a model of I/R after hemorrhagic shock [25], NAC preserved hepatic tissue. In a model of hepatic I/R associated with hepatectomy, enzymatic and morphological protective effects were observed in the liver after NAC treatment [26,27]. In a study in which NAC was compared to and associated with ischemic preconditioning, no synergistic or antagonistic effects were observed in hepatic or pulmonary tissue after hepatic I/R, indicating that the protective effects were similar [28].

In a study with rats fed a high fat diet (HFD) to induce NASH, it was observed that NAC (500 mg/kg/day) reduced the levels of total GSH and hepatic MDA to normal levels [29]. NAC (2 g/kg/day) also prevented many aspects of NASH progression by decreasing development of oxidative stress, but it was unable to block development of steatosis [30].

Although the use of NAC has been evaluated in several studies, which have demonstrated its beneficial effects in ameliorating the damages caused by oxidative stress and inflammation derived from NAFLD/NASH or I/R, there have been few studies on the effects of exogenous antioxidants in hepatic I/R models associated with steatohepatitis. Therefore, we decided to evaluate the performance of NAC in the treatment of inflammation, oxidative stress, and hepatic cell death in mice with inducible steatohepatitis submitted to hepatic I/R.

## 2. Results

### 2.1. Hepatic Enzymes

**Figure 1:**
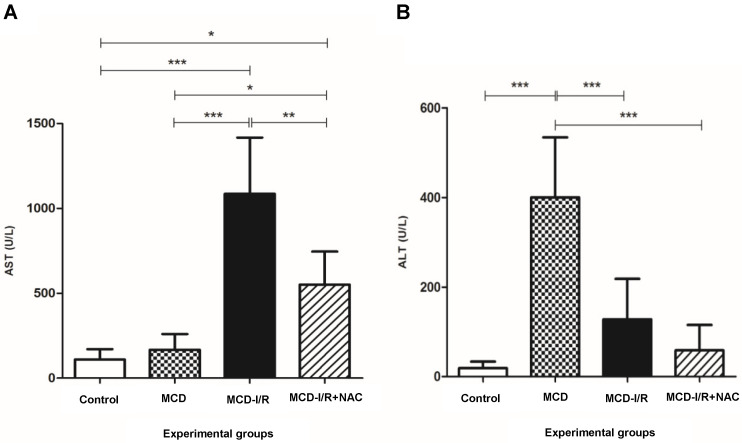
Levels of hepatic enzymes in the liver of normal mice and of animals submitted to ischemia/reperfusion (I/R) in the methionine- and choline-deficient (MCD) diet treated or not with *N*-acetylcysteine (NAC). The figure shows the levels of (**A**) aspartate aminotransferase (AST) and (**B**) alanine aminotransferase (ALT) (* *p* < 0.05, ** *p* < 0.01, and *** *p* < 0.001; N = 5–6; Bonferroni one-way ANOVA).

Aspartate aminotransferase (AST) levels were higher in methionine- and choline-deficient (MCD)-I/R and MCD-I/R+NAC groups than when compared to that of control and MCD groups. AST levels were lower in the MCD-I/R+NAC group compared to that of the MCD-I/R group (Figure 1A).

Alanine aminotransferase (ALT) levels were higher in the MCD group compared to that of the other groups. While the levels of both transaminases were reduced in the MCD-I/R+NAC group compared to that of the MCD-I/R group, only for AST was it significant (Figure 1A,B).

### 2.2. Oxidative Stress

**Figure 2:**
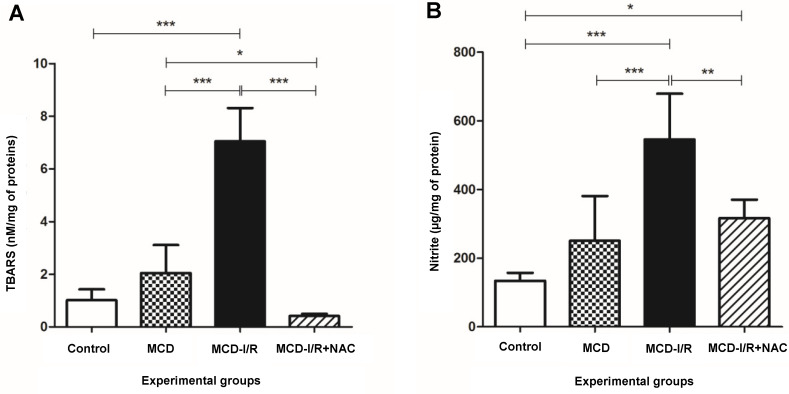
Concentrations of thiobarbituric acid reactive substances (TBARS) and nitrite in the liver of normal mice and of animals submitted to I/R in the MCD diet treated or not with *N*-acetylcysteine. The figure shows the levels of (**A**) TBARS and (**B**) nitrite (* *p* < 0.05, ** *p* < 0.01, and *** *p* < 0.001; N = 5–6; Bonferroni one-way ANOVA).

Oxidative stress from steatohepatitis and I/R was evidenced by the presence of lipid peroxidation and nitrite in hepatic tissue. The animals of the MCD-I/R group showed higher thiobarbituric acid reactive substances (TBARS) levels compared to that of the control and MCD groups. However, TBARS levels in the MCD-I/R+NAC group were lower than those in the MCD and MCD-I/R groups (Figure 2A).

The nitrite levels were higher in the MCD-I/R and MCD-I/R+NAC groups compared to that of the control group. However, as observed for TBARS levels, nitrite levels were lower in the MCD-I/R+NAC group compared to that of the MCD-I/R group (Figure 2B).

### 2.3. Antioxidant Enzyme Activities and Transcription Factor Nrf2

**Figure 3:**
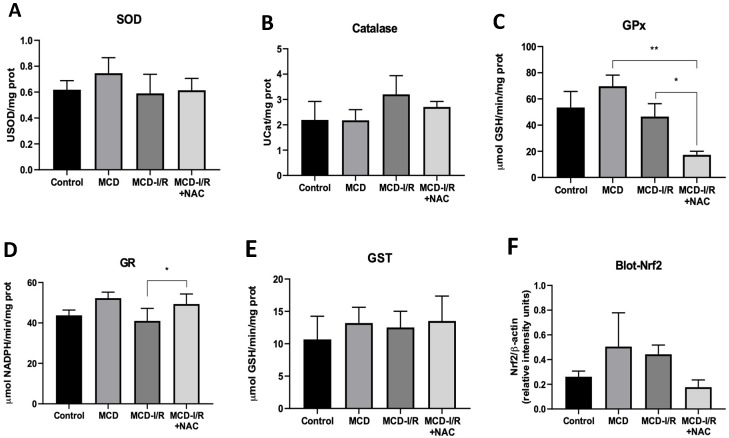
Activities of antioxidant enzymes and transcription factor Nrf2 in the liver of normal mice and of animals submitted to I/R in the MCD diet treated or not with *N*-acetylcysteine. This figure shows activity of (**A**) Superoxide dismutase, (**B**) catalase, (**C**) glutathione peroxidase, (**D**) glutathione reductase, and (**E**) glutathione S-transferase (* *p* < 0.05 vs. MCD-I/R; ** *p* < 0.01 vs. MCD; N = 5–6; Tukey one-way ANOVA), and (**F**) analysis of the densitometry of the Western blot evaluation of transcription factor Nrf2 (*p* < 0.08 vs. MCD-I/R; N = 4; one-way ANOVA).

It is possible to observe a non-significant increase in the activity of catalase in the MCD-I/R group (Figure 3B), suggesting a greater conversion of hydrogen peroxide into water. However, the activities of superoxide dismutase (Figure 3A) and glutathione S-transferase (Figure 3E) are similar among studied groups. A significant reduction in the activity of glutathione peroxidase (Figure 3C) was observed in the MCD-I/R+NAC group when compared to that of the MCD and MCD-I/R groups. It is also possible to observe an increase in the activity of glutathione reductase (Figure 3D) in the MCD-I/R+NAC group when compared to that of the MCD-I/R group.

The expression of the transcription factor Nrf2 (Figure 3F) was reduced in animals of the MCD-I/R+NAC group compared to that of the other groups, although it did not show a significant difference (*p* < 0.08 vs. MCD-I/R).

### 2.4. Hepatic Cytokines

**Figure 4:**
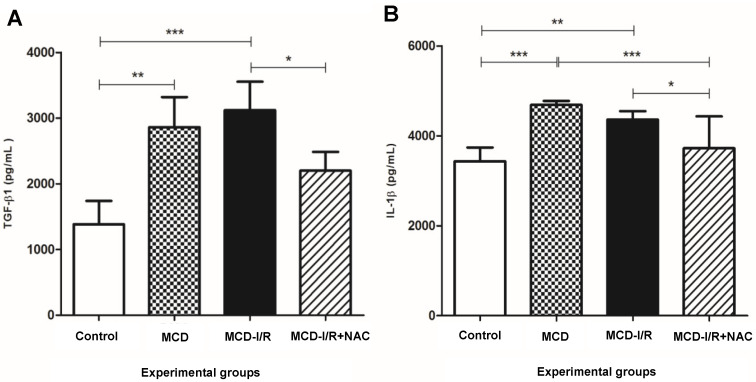
Concentrations of the pro-inflammatory cytokines in the liver of normal mice and of animals submitted to I/R in the MCD diet treated or not with *N*-acetylcysteine. The figure shows (**A**) cytokine IL-1β and (**B**) cytokine TGF-β1 (* *p* < 0.05, ** *p* < 0.01, and *** *p* < 0.001; N = 5–6; Bonferroni one-way ANOVA).

The levels of IL-1β and TGF-β1 were higher in the hepatic steatosis groups than those in the control group. IL-1β was also elevated in the MCD group compared to that of the MCD-I/R+NAC group. In addition, the MCD-I/R+NAC group showed lower levels of IL-1β and TGF-β1 than the MCD-I/R group (Figure 4A,B). The levels of IFN-γ in hepatic tissue were similar among the groups (control group: 276 ± 15 pg/mL, MCD group: 294 ± 26 pg/mL, MCD-I/R group: 223 ± 38 pg/mL, and MCD-I/R + NAC group: 314 ± 75 pg/mL).

### 2.5. Histology

**Figure 5:**
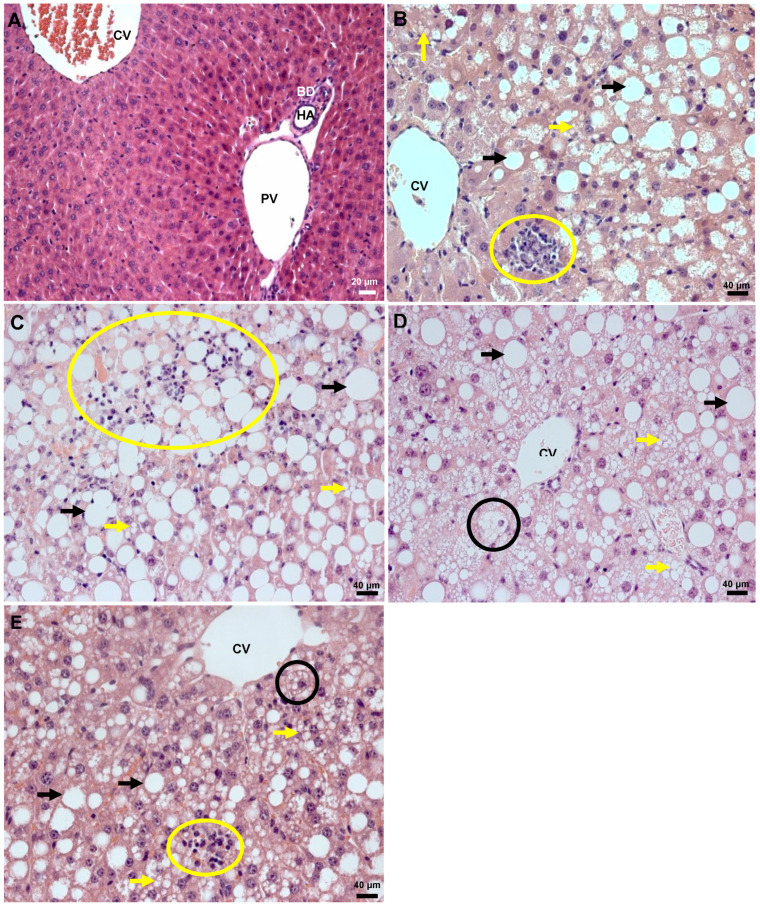
Histological changes in the liver of normal mice and of animals submitted to I/R in the MCD diet treated or not with *N*-acetylcysteine. This figure shows the (**A**) control group, (**B**) MCD group, (**C**,**D**) MCD-I/R group, and (**E**) MCD-I/R+NAC group. The black arrows show the presence of macrovesicular steatosis and the yellow arrows indicate microvesicular steatosis. The areas circled in yellow indicate the presence of inflammatory infiltration, and the areas circled in black show hepatocyte ballooning. Hepatic tissue was stained with hematoxylin and eosin (HE). CV = central vein; BD = bile duct; HA = hepatic artery; PV = portal vein. Magnification: 20× (**A**) and 40× (**B**–**E**).

Compared to the control group, the experimental groups showed the presence of fat vesicles and inflammatory foci, and differences in the morphological structures of hepatocytes (Table 1). The normal architecture of hepatic parenchyma was found in the control group (Figure 5A). The MCD group showed panacinar macrovesicular hepatic steatosis, with level 3 lobular inflammation (Figure 5B). The MCD-I/R group (Figure 5C), in addition to the characteristics mentioned above, presented level 1 hepatocyte ballooning (Figure 5D). In the MCD-I/R+NAC group, most of the mice presented level 3 periportal macrovesicular hepatic steatosis, and there was also a difference in the extent of the involved parenchyma but not in the degree of steatosis between MCD-I/R+NAC group mice and that of the MCD-I/R group mice. In addition, fewer inflammatory foci in hepatic tissue were observed in the MCD-I/R+NAC group compared to that of the MCD-I/R group, with only level 2 lobular inflammation and level 1 hepatocyte ballooning (Figure 5E).

### 2.6. Apoptosis

**Figure 6:**
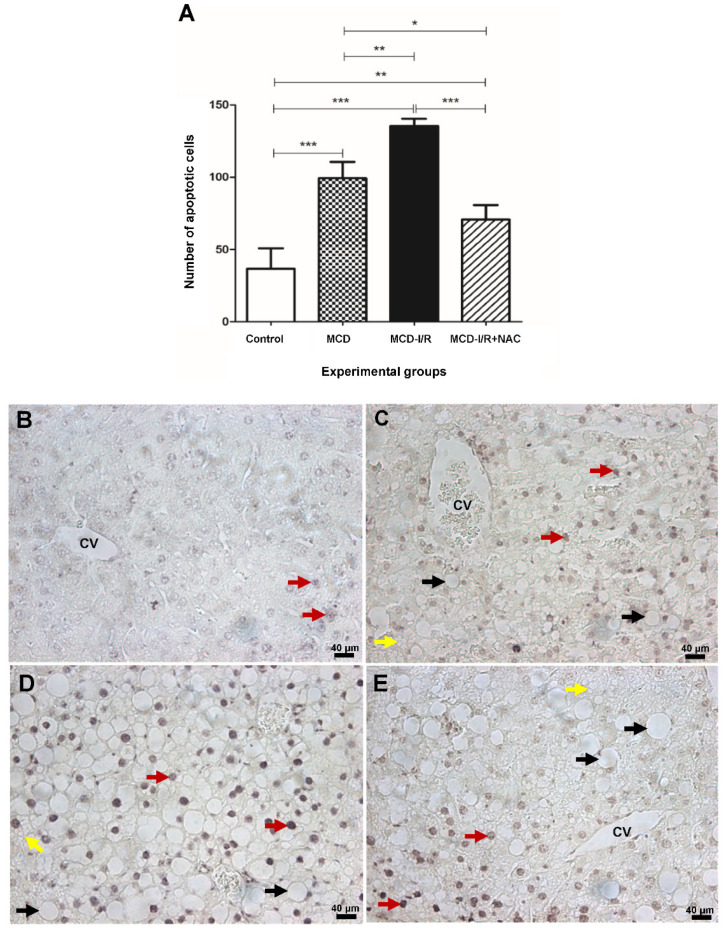
Apoptosis in mouse livers with steatohepatitis submitted to I/R and the effect of *N*-acetylcysteine on hepatic tissue. In (**A**), the results of statistical comparisons among the experimental groups are presented, where * *p* < 0.05, ** *p* < 0.01, and *** *p* < 0.001. The photomicrographs (magnification of 40×). (**B**) Control group. (**C**) MCD group. (**D**) MCD-I/R group. (**E**) MCD-I/R+NAC group. The red arrows indicate apoptotic cells detected by the TUNEL technique, the black arrows indicate the presence of macrovesicular steatosis, and the yellow arrows indicate microvesicular steatosis. CV = central vein.

Steatohepatitis and I/R resulted in apoptosis in hepatic tissue, although histopathological evaluation of the liver did not demonstrate the presence of necrosis. The MCD groups (Figure 6A,C–E) had more apoptotic cells than the control group (Figure 6B), and more apoptotic cells were observed in the MCD-I/R group than in the MCD and MCD-I/R+NAC groups (Figure 6C,D). NAC administration promoted a reduction in the number of apoptotic cells in the MCD-I/R+NAC group (Figure 6E) compared to that of the MCD and MCD-I/R groups (Figure 6C,D).

**Figure 7:**
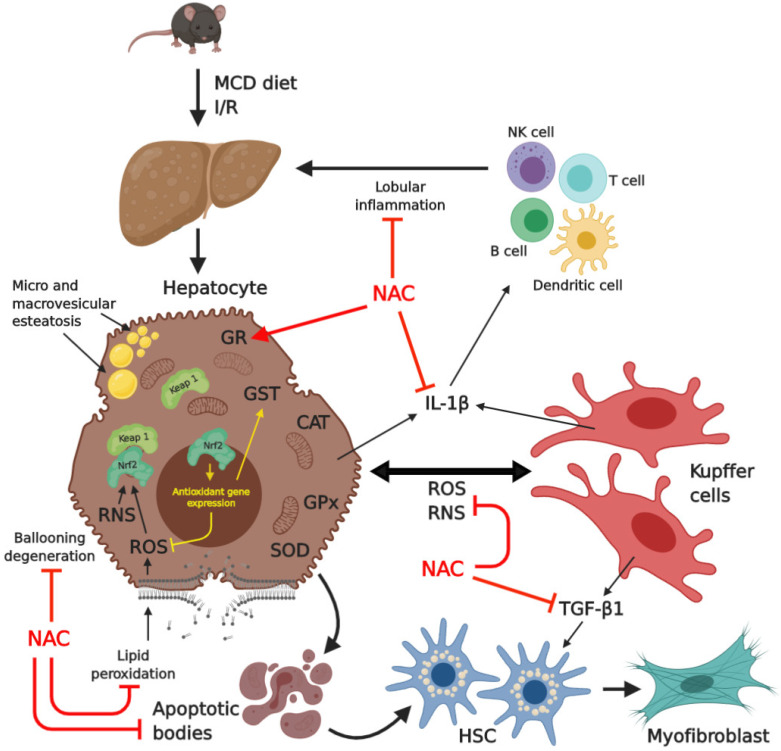
This is a schematic representation of all of the effects of *N*-acetylcysteine (NAC) in mice livers with MCD diet-induced steatohepatitis submitted to I/R injury. NAC reduced lipid peroxidation, RSN, IL-1β, TGF-β1, inflammatory foci, and apoptosis and increased glutathione reductase.

Summarizing the results found in this study, it was possible to observe that NAC showed a protective effect by increasing glutathione reductase and decreasing lipid peroxidation, the release of RNS, the number of apoptotic cells, tissue inflammation, and cytokines IL-1β and TGF-β1 (Figure 7).

## 3. Discussion

In our study, using a steatohepatitis model in mice submitted to hepatic I/R, NAC was able to alleviate oxidative stress by reducing lipid peroxidation and nitrite and by increasing glutathione reductase. In addition, NAC promoted a decrease in the number of apoptotic cells and in inflammation, according to the assessment of the inflammatory foci and the expression of the cytokines IL-1β and TGF-β1.

NAFLD is a very common disease in the world, showing a median estimated prevalence of 20% worldwide, especially in countries where sedentary behavior and high-calorie diets are usual. NAFLD is considered the hepatic manifestation of metabolic syndrome, being associated with increased obesity, dyslipidemia, and insulin resistance [31,32]. These conditions can aggravate situations that require surgery, either elective or emergency surgery. Therefore, the present study was designed to evaluate the potential of an exogenous antioxidant, NAC, to reduce the deleterious effects of oxidative stress, inflammation, and cell death associated with NAFLD and I/R, thus improving patient recovery.

In the current work, histopathological analysis of the liver with steatohepatitis submitted to hepatic I/R showed that NAC exerted beneficial effects on inflammation, since it reduced the presence of inflammatory foci. Previous studies corroborate this finding; however, the action of NAC in those studies was analyzed either in animals with NASH or liver I/R injury, but not in the two associated conditions [29].

Measuring serum AST and ALT concentrations is one of the most common ways to test for the presence of liver disease [33]. In our study, I/R injury associated with steatohepatitis promoted an augmentation in AST levels that was attenuated by NAC. With regard to ALT, significant increases in the serum level of this transaminase was observed only in mice with steatohepatitis compared to those of mice that did not suffer hepatic I/R injury. Wang et al. observed reduction in AST and ALT levels after administration of NAC in livers submitted to I/R [34]. In a model of NAFLD induced by MCD diet, the authors observed reduced levels of ALT after treatment with NAC in combination with resveratrol [35]. Nasiri et al. observed such reductions in ALT levels during liver regeneration in mice submitted to I/R [36]. In this study, the ALT levels increased within three hours of reperfusion and decreased to less than half within 24 h. After four days, ALT had returned to control levels, showing that the liver can regenerate rapidly over time. In our model, I/R injury associated with steatohepatitis decreased ALT levels drastically compared to the steatohepatitis group, and NAC was not able to show a significant improvement. Maybe hepatic regeneration capacity was compromised by the steatohepatitis, which implies inflammation and cell death.

NAFLD is a multifactorial disease with a complex pathogenesis. However, it is possible to highlight the accumulation of lipids in the hepatocytes associated with oxidative stress induced by I/R injury. This activates the cells of the immune system, inducing inflammation in the hepatic tissue through the production of inflammatory mediators, such as cytokines [37].

In our results, high concentrations of TBARS and nitrite were observed in the liver of animals that developed steatohepatitis and underwent I/R. However, administration of NAC promoted a marked reduction in TBARS and nitrite levels, showing the beneficial antioxidant effect of NAC in this experimental model with two concurrent injuries. Sun et al. showed that NAC alleviated hepatic injury caused by oxidative stress of the endoplasmic reticulum after I/R by reducing the concentrations of TBARS and ROS [38]. Hsieh et al., in turn, stated that NAC reduced TBARS levels and NO activity in livers submitted to I/R [39]. Baumgardner et al. observed the effect of NAC on the levels of TBARS in rats that developed NAFLD through total enteral nutrition with high fat levels [30]. In these studies, the reduction in oxidative stress promoted by NAC was verified in injury related to I/R or NAFLD. Fusai et al. observed that NAC improved damage in the hepatic tissue of rabbits submitted to warm liver I/R by reducing the high levels of ROS and RNS [40]. Nakano et al., in turn, showed the role of NAC in preventing I/R injury in the steatotic liver of rats by increasing the concentration of hepatic glutathione [41]. These studies corroborate our results regarding the reduction of ROS and RSN.

The use of NAC as an external antioxidant agent has already been demonstrated in other studies [34,42], in addition to being a substrate for cysteine and a precursor molecule of GSH. Demir and Inal-Erden demonstrated that NAC might be useful to ameliorate I/R injury in hepatic tissue by increasing the activities of the reduced glutathione-related enzymes [43]. Glutathione peroxidase is an enzyme that protect from oxidative stress, however, its level was reduced in the mice with steatohepatitis submitted to I/R and treated with NAC. This reduction could be explained by the fact that the NAC had enough time during the reperfusion (24 h) to reduce the free radicals, hence diminishing the gutathione peroxidase (GPx) synthesis. Yet, the increase in glutathione reductase (GR) activity in the treated group with NAC, when compared to that of the MCD-I/R group, indicates that there is an increase in the conversion to GSH, a good sign to show the anti-oxidant activity of NAC.

Some studies have demonstrated that NAC influences Nrf2 decrease in cases of liver damage [44,45]. Here, we demonstrated that Nrf2 expression decreased, but not statistically, and previous studies indicated that this transcription factor may be activated or not independently from antioxidant effects [46,47]. Taking into account the referred studies, the NAC action on the oxidative stress and inflammatory response in mice with steatohepatitis associated with I/R injury may occur regardless of Nrf2 activation.

Treatment with NAC was capable of ameliorating MCD-I/R, most likely due to the combination of its antioxidant [48] and anti-inflammatory [49,50] effects. This direct response appears to play a role in reducing lipid peroxidation, even with reduction in GPx activity, which may be a consequence of reduced Nrf2r expression. Increase in antioxidant activity has been associated with the NAFLD experimental condition as a physiological response and, as we showed, NAC treatment exerts protective effects [49], even considering that the ischemic-induced damage aggravated the chronical modulation of the oxidative stress, caused by MCD diet, and energetic unbalance. Further studies will be needed for a better comprehension of the effects of NAC on the mechanisms related to Nfr2 activity in these two associated conditions.

In our study, the increased expression of the IL-1β and TGF-β1 cytokines in mice with steatohepatitis associated with or without I/R injury was also attenuated by NAC. This finding demonstrates the ability of this exogenous antioxidant to reduce inflammation under the conditions evaluated. The expression of IFN-γ did not differ among the studied groups. Alexandropoulos et al. showed a beneficial effect of NAC on hepatic and renal injury caused by intestinal I/R in rats, as evidenced by reductions in the concentrations of IL-1β and other pro-inflammatory cytokines [51]. El-Lakkany et al. showed that combined use of NAC and metformin in livers with NAFLD reduces the concentrations of TGF-β, TNF-α, and other inflammatory mediators and decreases oxidative stress [52]. The decrease of these cytokines reduces the liver damage and the progression of hepatic fibrosis, since IL-1β is related to the amplification of the inflammatory process and contributes to the secretion of profibrogenic cytokine TGF-β, which promotes the activation of hepatic stellate cells. This activation, in turn, up-regulates the transcription inflammasome components and induces the collagen deposition that triggers liver fibrosis [37]. In our study, however, NAC alone was able to reduce the concentrations of both cytokines IL-1β and TGF-β1 in livers affected by these two associated conditions.

There is a resident population of natural killer (NK) and natural killer T (NKT) cells in livers, which respond to damage quickly. When these cells are active, they produce IFN-γ that can enhance neutrophil accumulation, tissue necrosis, synthesis of other pro-inflammatory cytokines, and generation of ROS and endoplasmic reticulum stress proteins in hepatocytes [39]. Lappas et al. [53] and Olthof et al. [54] demonstrated increased levels of IFN-γ after 72 and 60 min of hepatic ischemia followed by 2 and 6 h of reperfusion, respectively. Ellet et al., in turn, showed elevated levels of IFN-γ in steatotic livers submitted to total ischemia (35 min) and reperfusion (1 h) [55]. The longest or total ischemia times and analysis of liver samples shortly after reperfusion could justify the discrepancy in our results, in which the association of steatohepatitis with partial ischemia did not change the IFN-γ concentration in the liver in a significant way.

Oxidative stress and the release of inflammatory mediators in the liver parenchyma lead to cell death through necrosis or apoptosis [56,57,58]. In our study, there was an increase in the number of apoptotic cells in mice with or without steatohepatitis associated with hepatic I/R damage. However, NAC promoted a significant decrease in the number of apoptotic cells in mice with steatohepatitis submitted or not to I/R. This result corroborates a previous study, in which Shi et al. observed decreased apoptosis in the steatotic livers of rats treated with activated charcoal NAC microcapsules [59]. Wang et al. demonstrated a similar response in mice affected by liver injury I/R treated with NAC [34]. This protective effect of NAC in regard to cell death was demonstrated in our study in mice with both the referred conditions: steatohepatitis and I/R.

In summary, NAC ameliorated oxidative stress, inflammatory responses, and apoptosis associated with hepatic I/R injury in mice with diet-induced steatohepatitis by lowering hepatic lipid peroxidation, reactive nitrogen species, pro-inflammatory cytokines, inflammatory cells, and number of apoptotic cells and increasing glutathione reductase. These findings show the beneficial effects of NAC in the presence of these two associated conditions. However, further studies will be necessary to better clarify the mechanisms of NAC action on signaling via an endogenous antioxidant.

## 4. Materials and Methods

### 4.1. Animals

This research project was developed with the approval of the Committee on Ethics in Animal Use (CEUA)—FMUSP under process number 243/13.

C57BL/6J mice aged 8 weeks and weighing between 25 and 30 g were obtained from the Institutional Center of Animal Care—FMUSP, São Paulo, SP, Brazil. The animals were housed in polypropylene cages (40 × 30 × 25 cm) in groups of a maximum of three animals and with access to chow and water ad libitum, and adequate sanitary conditions were maintained. Steatohepatitis was induced by feeding an MCD diet for 30 days.

The animals were divided into four experimental groups: the control group, which was submitted only to anesthesia and laparotomy; the MCD group, which was fed an MCD diet and submitted only to anesthesia and laparotomy; the MCD-I/R group, which was fed an MCD diet and submitted to I/R; and the MCD-I/R+NAC group, which was fed an MCD diet, submitted to I/R, and treated with NAC (150 mg/kg, iv; Zambon S.p.A., Vicenza, Italy).

### 4.2. Surgical Procedures

The animals were treated with atropine (0.04 mg/kg, im; Ariston, São Paulo, SP, Brazil); ten minutes later, they were anesthetized with xylazine (10 mg/kg, im; Ceva Santé Animale, Paulínia, SP, Brazil) and cloridrate of dextrocetamine (70 mg/kg, im; Cristália, Itapira, SP, Brazil). In the MCD-I/R and MCD-I/R+NAC groups, the animals were submitted to 30 min ischemia followed by 24 h of reperfusion. NAC was administered to the MCD-I/R+NAC group in a single dose 15 min before ischemia. After 24 h of reperfusion, the animals were again anesthetized for blood collection and surgical removal of the liver.

### 4.3. Hepatic Enzymes

Blood was obtained by cardiac puncture and centrifuged at 956 *g* for 15 min at 4 °C, and the serum was frozen in liquid nitrogen and kept in a −80 °C freezer for later analysis of ALT and AST.

### 4.4. Oxidative Stress

#### 4.4.1. TBARS

For this analysis, liver tissue was homogenized with phosphate buffer (pH 7.2). An aliquot of the tissue supernatant was used for determination of total proteins by the Bradford method. The samples were diluted 1:10, mixed with 300 μL of Bradford reagent, and read in a spectrophotometer with a 595 nm filter. After quantification of total proteins, TBARS were assessed. To the liver samples, diluted in the same ratio as above, 250 μL of 17.5% trichloroacetic acid (TCA) and 250 μL of 0.6% thiobarbituric acid (TBA) were added, and the mixtures were incubated for 15 min in a water bath at 95 °C. Then, 250 μL of 70% TCA was added, and the mixtures were incubated for 20 min at 4 °C. The absorbance of the final solution was then measured in a spectrophotometer with a 534 nm filter.

#### 4.4.2. Nitrite

Liver samples were homogenized in phosphate buffer (pH 7.2). The limit of detection for this method was 1.0 μM nitrite. Fifty microliters of each sample was placed in a 96-well plate along with serial dilutions for a standard curve. Subsequently, 50 μL of Griess reagent, previously prepared with equal volumes of component A (*N*-(1-naphthyl) ethylenediamine dihydrochloride) and component B (sulfanilic acid), was added to each well containing a sample or a dilution for the standard curve, and the plate was incubated for one hour at room temperature. A photometric reference sample was prepared by mixing 50 μL of Griess reagent and 50 μL of deionized water. After incubation, the absorbance of the final solution was then measured in a spectrophotometer with a 534 nm filter.

#### 4.4.3. Sample Preparation for Measurement of Antioxidant Enzymes

The samples were weighed, homogenized in phosphate buffer (50 mM; pH 7.0) in the proportion of 1:10, centrifuged (10,000 rpm, 4 °C, 10 min), and the supernatant was separated for enzymatic measurement.

##### Determination of SOD Activity

Superoxide dismutase (SOD) activity was determined by the formation of the XTT-formazan product [60]. The measured reaction occurs between xanthine, xanthine oxidase, and SOD, generating the superoxide radical anion (O_2_^•−^). This in turn reduces the XTT reagent (Sigma; St. Louis, MO, USA) to the XTT-formazan product, which absorbs light at 470 nm o-dianisidine (OD). SOD hijacks O_2_^•−^ and reduces the formation of the XTT-formazan product. The result was expressed as SOD units (USOD)/mg of protein. The SOD unit was defined as the amount of SOD capable of transforming 1 μmol/min of O_2_.

##### Determination of CAT Activity

Catalase (CAT) activity was performed after diluting the sample (1:100) in 50 mM phosphate buffer. The method involves two reactions: (1st) H_2_O_2_ (10 nM) undergoes dismutation by tissue catalase for 10 min at room temperature. This reaction is stopped by the addition of NaN_3_ (1 mM); (2nd) the remaining H_2_O_2_ is determined by oxidation of the o-dianisidine reagent (OD; 0.167 mg/mL; Sigma; St. Louis, MO, USA) in a reaction catalyzed by the enzyme peroxidase HRP (horse radish peroxidase; 0.095 mg/mL; Sigma; St. Louis, MO, USA), at pH 6.0. The speed of the o-dianisidine oxidation product was monitored by the increase in absorbance at 460 nm (Spectra max Plus 384, Molecular Devices Inc.; Sunnyvale, CA, USA) for 10 min. In order to inactivate the catalase (reaction blank), supernatants were incubated at 60 °C for 2 h. The catalase activity value was calculated from the maximum speed per minute of each reaction and extrapolated on the H_2_O_2_ curve. The standard H_2_O_2_ curve (8820–11.3 μM) was performed and the results were expressed in catalase units (UCAT)/mg protein. A catalase unit was defined as the degradation of 1 µmol of H_2_O_2_ min^−1^ at 25 °C.

##### Determination of GPx Activity

GPx activity was determined by indirect measurement of GPx activity, through a reaction associated with glutathione reductase (GR). Oxidized glutathione (GSSG), produced by reduction via hydroperoxides by GPx, was recycled to generate its reduced state by GR (Sigma; St. Louis, MO, USA) and NADPH (Sigma; St. Louis, MO, USA) [61]. The substrate used was tert-butyl hydroperoxide. The oxidation of NADPH to NADP+ was accompanied by a decrease in absorbance at 340 nm at 37 °C. The samples were analyzed in duplicate and expressed as μmol GSH/min/mg protein.

##### Determination of GR Activity

This detection was based on a direct measure of GR activity, which used NADPH as a cofactor in the reduction of GSSG in GSH. The oxidative reaction from NADPH to NADP^+^ was measured via absorbance decay under o-dianisidine (OD) equal to 340 nm at 37 °C [62]. The samples were analyzed in duplicate and expressed as μmol NADPH/min/mg protein.

##### Determination of GST Activity

The GST activity was based on the generation of a complex between GSH and 1-chloro-2,4-dinitrobenzene (CDNB; Sigma; St. Louis, MO, USA), catalyzed by GST. The increase in absorbance was directly proportional to the GST activity in the sample [63], which was measured under o-dianisidine (OD) equal to 340 nm for 30 min at a temperature of 25 °C. The samples were analyzed in duplicate and the results were expressed as μmol GSH conjugate/min/mg protein.

##### Western Blotting

Samples were prepared and the liver was homogenized in lysis buffer (HEPES 10 mM; MgCl2 1.5 mM; KCl 10 mM; DTT 0.5 mM; PMSF 0.5 mM; leupeptin 2 µg/mL; antipain 2 µg/mL) following the descriptions of [64]. After centrifugation of the homogenate (11,000 *g*, 20 min; 4 °C) the pellet was resuspended in 300 μL of extraction buffer (20 mM HEPES; 1.5 mM MgCl_2_; 300 mM NaCl; 500 mM EDTA; Glycerol 25%; 2 µg/mL leupeptin; 2 µg/mL antipain; 0.5 mM PMSF; 3 mM orthovonadate; 0.5 mM DTT), stored on ice for 30 min, and centrifuged at 20,000 *g* for 5 min. The supernatant containing the nuclear extract was separated for Western blot analysis. The Western blot assay for Nrf2 was performed according to Munhoz et al. [65].

The nuclear extract (30 µg) was applied to the 10% polyacrylamide gel (acrylamide/bisacrylamide (5:1)). For electrophoresis, the tris buffer (25 mM Tris; 192 mM Glycine; 1% SDS) was used. The gel was subjected to electrophoresis for 2 h at 150 V, followed by transfer to a nitrocellulose membrane (#1620112; BioRad; Hercules, CA, USA) for 1 h 30 min at 35 V. The membranes had non-specific sites blocked with 3% albumin (Santa Cruz Biotechnology; Santa Cruz, CA, USA) and then were incubated with the primary anti-Nrf2 antibody (1: 1000; MAB3925; R&D Systems; Minneapolis, MN, USA) overnight at 18 °C. After the incubation period, the membranes were incubated with secondary antibody conjugated to horseradish peroxidase (HRP; 1: 3000; #1705047; BioRad; Hercules, CA, USA) for 2 h at room temperature, and the signal was obtained with Luminata Forte Western HRP (Merck Millipore; Darmstadt, Hessen, Germany) using the ChemiDoc system (Bio-Rad; Hercules, CA, USA). The relative density of the bands was normalized to the values of β-Actin (1:3000; 8H10D10; Cell Signaling Technology, Danvers, Massachusetts, USA).

### 4.5. Hepatic Cytokines

For evaluation of cytokines in hepatic tissue, the samples were homogenized in PHEM buffer (pH 7.2) and measured by the ELISA method. Plates (96-well) were sensitized with specific capture antibodies for each cytokine (IL-1β, TGF-β, and IFN-γ), washed with phosphate-buffered saline (PBS) and 0.05% Tween 20, and then blocked with PBS and 10% fetal bovine serum (FBS).

The plates were incubated with the homogenate supernatants for one hour at room temperature and then incubated with a biotinylated detection antibody and a streptavidin-peroxidase conjugate. Finally, the plates were developed with tetramethylbenzidine for 15 to 30 min at room temperature with protection from light. The reaction was interrupted with a 2 N sulfuric acid solution, and the absorbance was read in a spectrophotometer with a 450 nm filter.

### 4.6. Histology

Left lateral hepatic lobe samples were fixed in 10% formol-saline, embedded in paraffin, and sectioned at a thickness of 4 μm. The sections were stained with hematoxylin and eosin (HE) and evaluated by a blinded examiner, according to the histological parameters described by Kleiner et al. (2005) [66]. Briefly, this score is defined as a sum of the scores for steatosis (0–3), lobular inflammation (0–3), and ballooning (0–2); thus, ranging from 0 to 8.

### 4.7. Apoptosis

The presence of apoptotic cells was investigated by terminal deoxynucleotidyl transferase (TdT) dUTP nick-end labeling (TUNEL) assay in 4 μm thick histological sections. An In Situ Cell Death Detection Kit, POD (Roche Applied Science, Penzberg, Upper Bavaria, Germany) was used. The sections were dewaxed in xylol and immersed in absolute alcohol and decreasing concentrations of alcohol (95%, 90%, 80%, 70%, and 50%). Then, the sections were washed in PBS (pH 7.2), incubated in k protein solution (20 μg/mL), washed in distilled water, and incubated in 3% hydrogen peroxide (H_2_O_2_) in neat methanol *v*/*v*. Then, the sections were blocked with 1% bovine serum albumin (BSA) and 20% FBS in 0.1 M Tris buffer (pH 7.5). The sections were then permeabilized with 0.2% Triton X-100 in sodium citrate buffer (pH 6.0), incubated in labeling solution (from the POD kit), washed with PBS, and incubated with converter solution (from the POD kit). Finally, the sections were visualized with a DAB/nickel solution, and the results were analyzed by an evaluator who was unaware of the group to which each section belonged.

### 4.8. Statistics

The levels of AST, ALT, TBARS, IL-1β, IFN-γ, SOD, CAT, GPx, GR, and GST are expressed as the mean ± standard deviation. The levels of TGF-β and nitrite and the numbers of apoptotic cells are expressed as the median (interquartile range). For statistical analysis of the results, one-way ANOVA and the Bonferroni and Tukey post-hoc tests or ANOVA for nonparametric data (Kruskal–Wallis test) and the Dunn’s post hoc test were applied. GraphPad Prism 5 software was used for statistical analysis. In all tests, the significance level was set at 5% (*p* < 0.05).

## Figures and Tables

**Table 1:** Liver histological aspects of normal mice and of animals submitted to I/R in the MCD diet treated or not with *N*-acetylcysteine (NAC). NAFLD: nonalcoholic fatty liver disease; NASH: nonalcoholic steatohepatitis.

Histologic Feature of NAFLD	Category	% Responses in the Groups (N = 4–6, per Group)			
		Control	MCD	MCD-I/R	MCD-I/R+NAC
Steatosis Grade	<5%	25%			
	5–33%	50%			
	>33–66%	25%	20%		25%
	>66%		80%	100%	75%
Location	Periportal (zone 1)	100%			75%
	Panacinar		100%	100%	25%
Microvesicular steatosis	Present	100%	100%	100%	100%
Macrovesicular steatosis	Present	0%	100%	100%	100%
Lobular inflammation	No foci	25%			
	<2 foci	25%			
	2–4 foci	50%			100%
	>4 foci		100%	100%	
Ballooning degeneration	None	100%	80%	66%	75%
	Few	0%	20%	34%	25%
NASH	Present	0%	100%	100%	100%

## Abbreviations

ALT	Alanine aminotransferase
ANOVA	Analysis of variance
ARE	Antioxidant response element
AST	Aspartate aminotransferase
BSA	Bovine serum albumin
CAT	Catalase
CEUA	Committee on ethics in animal use
DAB	Diaminobenzidine
ELISA	Enzyme-linked immunosorbent assay
FBS	Fetal bovine serum
FMUSP	Faculdade de Medicina da Universidade de São Paulo
GHS	Reduced glutathione
GPx	Glutathione peroxidase
GR	Glutathione reductase
GST	Glutathione S-transferase
HE	Hematoxylin and eosin
HFD	High fat diet
HSC	Hepatic stellate cells
ICHC-FMUSP	Instituto Central do Hospital das Clínicas-FMUSP
IFN-γ	Interferon gamma
IL-1β	Interleukin 1 beta
I/R	Ischemia/reperfusion
Keap1	Kelch-like ECH-associated protein 1
MCD	methionine- and choline-deficient
MCD-I/R+NAC	methionine- and choline-deficient with ischemia/reperfusion plus *N*-acetylcysteine
MCD-I/R	methionine- and choline-deficient with ischemia/reperfusion
NAC	*N*-acetylcysteine
MDA	Malondialdehyde
NAFLD	Nonalcoholic fatty liver disease
NASH	Nonalcoholic steatohepatitis
NF-κB	Nuclear factor κ-light-chain enhancer activated B cells
NK	Natural killer
Nrf2	Nuclear factor erythroid-2-related factor 2
PBS	Phosphate-buffered saline
RNS	Reactive nitrogen substances
ROS	Reactive oxygen substances
SOD	Superoxide dismutase
TBA	Thiobarbituric acid
TBARS	Thiobarbituric acid reactive substances
TCA	Trichloroacetic acid
TGF-β1	Transforming growth factor beta 1
TNF-α	Tumor necrosis factor alpha
TUNEL	Terminal deoxynucleotidyl transferase dUTP nick end labeling
USP	Universidade de São Paulo
